# Managing Refractory Hypoxemia in Acute Respiratory Distress Syndrome Obese Patients with Veno-Venous Extra-Corporeal Membrane Oxygenation: A Narrative Review

**DOI:** 10.3390/jcm14051653

**Published:** 2025-02-28

**Authors:** Arnaud Robert, Patrick M. Honoré, Pierre Bulpa, Isabelle Michaux

**Affiliations:** 1 Department of ICU, Centre Hospitalier Universitaire Université Catholique de Louvain, Mont-Godinne, 5530 Yvoir, Belgium; 2 The Faculty of Medicine, Experimental Research Laboratory Institute of the Catholic Louvain Medical School, 1200 Brussels, Belgium

**Keywords:** ARDS, refractory hypoxemia, vvECMO, prone positioning, obesity

## Abstract

Veno-venous extracorporeal membrane oxygenation (vvECMO) is a life-saving intervention for severe respiratory failure unresponsive to conventional therapies. However, managing refractory hypoxemia in morbidly obese patients poses significant challenges due to the unique physiological characteristics of this population, including hyperdynamic circulation, elevated cardiac output, and increased oxygen consumption. These factors can limit the effectiveness of vvECMO by diluting arterial oxygen content and complicating oxygen delivery. Refractory hypoxemia in obese patients supported by vvECMO often stems from an imbalance between ECMO blood flow and cardiac output. Hyperdynamic circulation exacerbates the recirculation of oxygenated blood and impairs the efficiency of oxygen transfer. To address these challenges, a stepwise, individualized approach is essential. Strategies to reduce oxygen consumption include deep sedation, neuromuscular blockade, and temperature control. Cardiac output modulation can be achieved through beta-blockers and cautious therapeutic hypothermia. Optimizing oxygen delivery involves improving residual lung function; high positive end-expiratory pressure ventilation guided by esophageal pressure monitoring; prone positioning; and adjustments to the ECMO circuit, such as using dual oxygenators, larger membranes, or additional drainage cannulas. This review highlights the interplay of physiological adaptations and technical innovations required to overcome the challenges of managing refractory hypoxemia in obese patients during vvECMO. By addressing the complexities of high cardiac output and obesity, clinicians can enhance the effectiveness of vvECMO and improve outcomes for this high-risk population.

## 1. Introduction

Veno-venous extracorporeal membrane oxygenation (vvECMO) has emerged as a life-saving intervention for patients with acute respiratory distress syndrome (ARDS) [1] with severe respiratory failure who are unresponsive to conventional therapies. Emerging data (EOLIA [2] and CESAR [3] trial) and the COVID-19 pandemic are changing mentalities toward vvECMO use in ARDS. As obesity prevalence has risen in the general population over the past decades, including among critically ill patients—now accounting for up to 20% of intensive care unit (ICU) cases [4]—ICU physicians considering ECMO should enhance their understanding of the specific challenges and considerations associated with this technique in obese and super-obese patients. Current guidelines recommend that clinicians consider vvECMO cannulation when EOLIA criteria are met [5]. Table 1 summarize the EOLIA criteria for ECMO initiation and the classical contraincations.

Although obesity is associated with an overall increased risk of morbidity and mortality in the general population, critically ill obese and super-obese patients (body mass index [BMI] > 40 kg/m^2^) are not showing increases in mortality during ICU stay [6] but might be associated with longer ICU stay and longer duration of mechanical ventilation [6,7] among survivors. These contradictory findings have been referred to as “the obesity paradox” [4,8]. Similar findings for ARDS obese patients [9] underscore the resilience of this population in severe respiratory failure. Concerns regarding the use of ECMO in obese patients include difficult vascular access for percutaneous cannulation technique, high cardiac output and blood volume, and an increased risk of cannula-related infections. However, despite these concerns, recent studies have shown that—consistent with previous data—obesity in patients on vv-ECMO may be associated with an increased survival rate [10,11]. Nonetheless, in the EOLIA study, patients with a BMI greater than 45 kg/m^2^ were excluded [2]. Despite the small sample size (12 patients) in their study, Swol et al. [12] further demonstrated the feasibility of using vv-ECMO in obese and super-obese patients. Their study included surgical patients with hypercapnic respiratory failure associated with a mean BMI of 47.9 kg/m^2^, with the highest BMI reaching 88 kg/m^2^. They also confirmed the feasibility of percutaneous cannulation and reported a 50% survival rate, which was comparable to the outcomes in the EOLIA and CESAR trials. Consequently, the use of vvECMO in that population can no longer be viewed as a contraindication or predictor of poor outcomes and may even be strongly considered. Nevertheless, in this particular population, the application of vvECMO requires particular management and technical nuances [9,13].

In obese patient, ARDS is frequently characterized by elevated cardiac output (CO) [14], elevated total blood volume [15], increased oxygen consumption [16], and impaired pulmonary mechanic resulting in high rates of atelectasis [17]. All of these factors can contribute to the development of refractory hypoxemia during vvECMO support. The hyperdynamic circulation and elevated blood volume necessary in obese patients in response both to the need to perfuse the additional mass and to increase baseline oxygen consumption dilutes arterial oxygen content, creating a scenario where even maximal ECMO flow may fail to meet metabolic demand [16].

**Figure 1:**
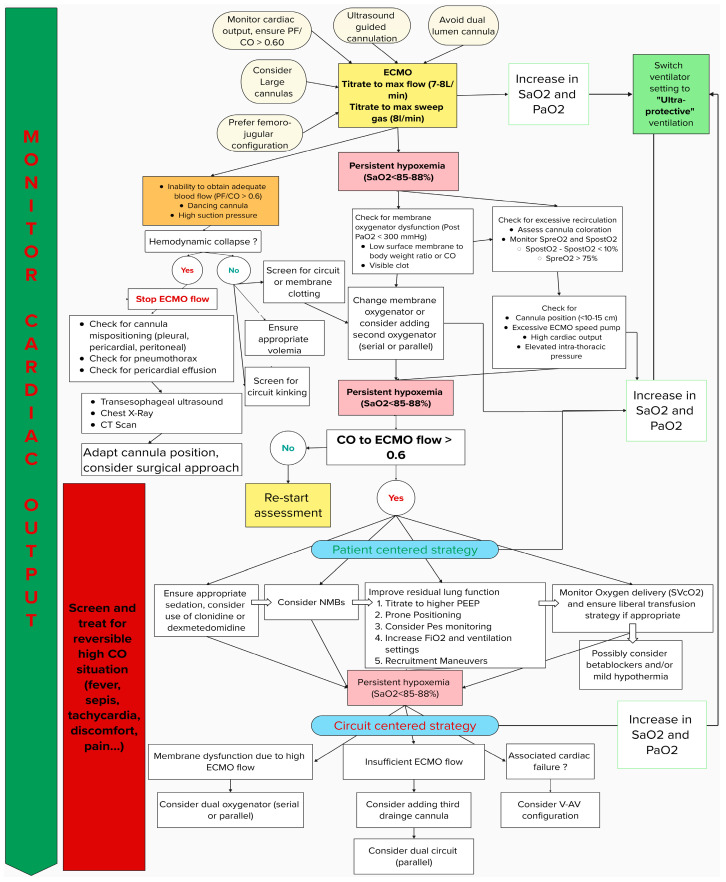
Flowchart of stepwise management for refractory hypoxemia in ARDS vvECMO among obese patients.

This review explores the physiological rationale, clinical strategies, and technical innovations involved in optimizing vvECMO for morbidly obese patients. As a practical reference, a flowchart summarizing the clinical strategies and providing a stepwise management approach is included to guide the implementation of these techniques in clinical practice (Figure 1). By understanding the interplay of these factors, clinicians can better navigate the challenges of managing refractory hypoxemia in this high-risk population and ensure that the promise of vvECMO is fully realized.

## 2. Data Collection

A comprehensive literature search was conducted using PubMed, Google Scholar, and Scopus to identify relevant articles on managing refractory hypoxemia in obese patients with ARDS using veno-venous extracorporeal membrane oxygenation (VV-ECMO). The research did not impose a date restriction; however, emphasis was placed on the most recent studies, and the language was limited to English and French articles.

The inclusion criteria were as follows: studies and/or systematic reviews/meta-analyses involving patients with ARDS receiving vvECMO that included obese patients. Use of veno-arterial ECMO was included only if no data were found for vvECMO. When such studies were unavailable, those examining ARDS in obese patients, regardless of ECMO use, were considered. If no data were found on obesity in ARDS, general ARDS studies were selected, and subgroup analyses of obese patients were extracted when possible. Reviews and expert opinions were excluded except for validated guidelines from scientific society. Finally, “Similar articles” and “Cited by” sections in PubMed Central as well as the references of included papers were screened for additional material not found in the initial literature research.

This approach ensured the inclusion of the most relevant and comprehensive data on the topic.

## 3. Physiological Explanation of Refractory Hypoxemia in vvECMO: Inadequacy of ECMO Blood Flow and Cardiac Output (CO)

Although permissive hypoxemia was initially thought to improve survival [18], recent studies have shown that lower is not always better. Refractory hypoxemia can be defined as the impossibility to achieve an SpO_2_ of 88–95% [19] or PaO_2_ > 60 mmHg [20]. This can occur immediately after cannulation or at any time during vvECMO support. These cutoff values are commonly used and recommended [1,21], as lower oxygen saturation has been linked to increased mortality and/or morbidity (e.g., mesenteric ischemia) [22] in critically ill patients with ARDS. Conversely, higher SpO_2_ levels have not been linked to improved survival [23,24], even in COVID-19-induced ARDS [25].

Before diagnosing refractory hypoxemia during vvECMO, technical issues such as a kinked circuit, recirculation, cannula misplacement, or membrane and pump dysfunction must be ruled out first. Once these factors are addressed, refractory hypoxemia can generally be attributed to an imbalance between ECMO flow and the patient’s CO, where the oxygen delivery capacity of the circuit fails to meet the physiological demands of the patient. In vvECMO, part of the venous return goes through the ECMO circuit and is oxygenated to 100%, then returned to the right atrium. The rest (patient venous return), with 60–80% of venous saturation, passes through the pulmonary circulation unaltered. These flows (ECMO and patient’s flow) mix in the right atrium and ventricle, move through the lungs, and determine arterial blood saturation, which is typically 80–90%. ECMO flow must be adjusted relative to total venous return (cardiac output) to optimize systemic oxygen delivery [5].

This can be synthesized in a formula determining arterial oxygen saturation (SaO_2_) associated with vvECMO [26]:

SaO_2_~S_PA_O_2_ = (EF/CO)SmO_2_ + (1 − EF/CO)SvO_2_

             + 0.01PmO_2_ + RLF

             where EF = (1 − R)PF

where:

**S_PA_O_2_** is oxygen saturation in the pulmonary artery (%);
**EF** is effective flow, the fraction of pump flow oxygenated by ECMO (L/min);
**SmO_2_** is oxygen saturation in the blood exiting the oxygenator (membrane; %);
**CO** is cardiac output (L/min);
**SvO_2_** is oxygen saturation in mixed venous blood (%);
**PmO_2_** is partial pressure of oxygen in the blood exiting the oxygenator (membrane; %);
**RLF** is residual lung function;
**R** is recirculation rate;
**PF** is pump flow.
The term **_0_._01_PmO_2_** (in %) is a numerical estimation of the increase in SaO_2_ due to dissolved oxygen in the blood exiting the oxygenator.

This formula indicates that higher ECMO flow results in higher arterial saturation (SaO_2_), while higher cardiac output results in decreased SaO_2_. High recirculation also reduces SaO_2_, and improved residual lung function will improve SaO_2_. This has been demonstrated in clinical studies. In ARDS cases, Schmidt et al. [27] showed that ECMO flow below 60% of the patient’s cardiac output usually results in SaO_2_ inferior to 90%.

Thus, central to this issue is the inadequacy of ECMO blood flow relative to the patient’s cardiac output, a dynamic interplay with significant physiological implications.

Patients may exhibit a hyperdynamic circulatory state with elevated CO in conditions like obesity but also liver failure, pregnancy, hyperthyroidism, fever, systemic inflammatory responses (SIRS) or septic shock, arteriovenous malformations, Paget’s disease, myeloproliferative disorders, or even general agitation [28]. These conditions can exacerbate the imbalance, as the fraction of deoxygenated blood bypassing the ECMO circuit increases unless the ECMO flow is proportionally escalated. Screening for these conditions throughout the ECMO course is essential and should prompt a thorough assessment of cardiac output.

Additionally, elevated CO in hyperdynamic states makes it even more challenging to match ECMO blood flow to metabolic demands. Conditions associated with high cardiac output often coincide with increased metabolic demands, heightened oxygen consumption [29], and greater CO_2_ production, which can ultimately lead to tissue hypoxia and lactic acidosis.

## 4. Clinical Management

Assessment of hypoxemia should start directly upon cannulation. Initially, the sweep gas flow should be carefully titrated over ECMO blood flow (usually up to 7–8 L/min), and the membrane fraction of oxygen (FmO_2_) should initially be set to 100% to ensure optimal oxygenation [26]. Before escalating therapy to improve oxygen saturation, clinicians should address common pitfalls of vvECMO management. If inadequate resaturation or if desaturation occurs immediately after cannulation, the ECMO team must screen for complications linked to ECMO cannulation such as hemothorax, pneumothorax, or derecruitment causing atelectasis. Therefore, chest X-ray should be performed directly after cannulation in order to rule out these complications and to additionally address the position of the cannulas [30]. Meanwhile, clinicians should follow a stepwise approach, as most cases of persistent hypoxemia post-ECMO cannulation are due to technical issues that can be easily evaluated. Key parameters to evaluate sequentially include assessment of ECMO blood flow, recirculation, and membrane dysfunction.

### Technical Issues

*ECMO blood flow*: ECMO blood flow should be titrated until optimal oxygen saturation or maximal ECMO capacity (usually up to 7–8 L depending on manufacturer) is obtained. If up titration of ECMO blood flow cannot be obtained, clinicians should raise concerns about cannulation misplacement (sus-hepatic vein, retroperitoneum in case of femoral cannulation, and pleural or pericardial space in case of jugular cannulation). This will often result in rapid hemodynamic deterioration and may ultimately lead to patient death. In such cases, the ECMO circuit should be stopped, and the ECMO cannula position should be assessed through adequate imaging (transesophageal ultrasound, chest X-ray, or CT scan depending on the severity of the patient and availability), and the surgical team should be advised if necessary. If adequate position of the cannulas is confirmed, the clinician should assess for hypovolemia and promptly initiate fluid resuscitation. Furthermore, kinking of the circuit can be a classic cause of inappropriate ECMO flow and should be ruled out quickly. Finally, inappropriate cannula purging during placement may result in massive clotting of the cannulas, prompting immediate replacement to avoid a massive pulmonary or air embolism.

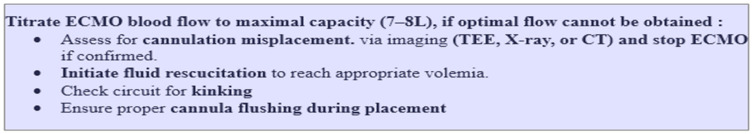



*Recirculation:* At high ECMO flows, the return of oxygenated blood through the ECMO circuit can inadvertently mix with the drainage blood at the cannula site. This reduces the effective oxygen transfer of the circuit, as already oxygenated blood is reoxygenated, leaving systemic venous blood inadequately addressed. This can be easily and rapidly assessed by first examining the blood color in both cannulas [31]; a similar reddish color could indicate significant recirculation. This can be evaluated also by arterial gas samples from the venous and arterial cannulas.

Excessive recirculation can be detected based on high SvO_2_ (usually > 75%) and minimal saturation difference (less than 10%) between pre-membrane saturation and post-membrane saturation. Additionally, recirculation fraction can be calculated upon this formula and should be less than 20–30% [32].

S_pré_Ox − SVO_2_/S_Post_Ox − SVO_2_

In a study by Pooth et al. [33], the authors showed that excessive recirculation often occurs due to the close proximity of the drainage and return cannulas, high ECMO blood flow, and low cardiac output [32]. BMI was not associated with a high recirculation ratio [32]. Certain configurations of cannulation (femoro-femoral) were associated with more recirculation than femoro-jugular or dual lumen cannulas and thus probably need to be avoided. The ideal range between the cannulas should be between 10 to 15 cm [26]. Dual lumen cannulas (e.g., Avalon cannulas) are known to reduce the risk of recirculation [33] due to their placement, with drainage orifices positioned in both superior and inferior vena cava, while the orifice reinjection jet is oriented directly toward the tricuspid valve. As illustrated, this precise placement requires an experienced clinician, as there is a steep learning curve associated with this technique [34]. Moreover, dual lumen cannulas do not permit high flow rates [35]; therefore, they should not be considered a viable alternative in cases of refractory hypoxemia due to high cardiac output. Other factors influencing recirculation are a high-speed pump, high CO to Q_ECMO_ ratio, and high intrathoracic pressure [32].

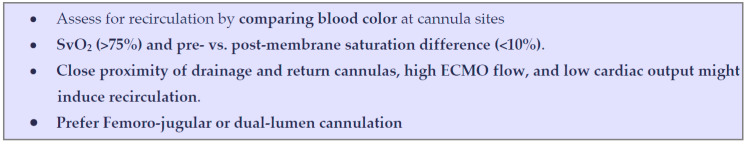



*Inadequate ECMO membrane efficiency or membrane dysfunction:* At 100% FmO_2_ and max sweep gas, post-membrane PaO_2_ should normally be higher than 300 mmHg [31]. If this is not the case, clinicians should assess for membrane dysfunction. First, screening for membrane clotting due to inappropriate cannulation is essential, followed by regular monitoring for increased pre-membrane pressure, visible blood clots, and altered post-membrane PaO_2_ [5,31]. Additionally, ECMO oxygenators have a limited capacity for oxygen transfer, which depends on the surface area of the oxygenator and the blood’s transit time through it. At high flow rates, the reduced transit time can limit oxygen diffusion, worsening the mismatch between oxygen supply and demand. The appropriate surface membrane should be chosen according to the patient’s morphology and CO. ECMO membrane surfaces vary from 0.75 m^2^ to 2.5 m^2^; a high surface membrane might be necessary in cases of morbidly obese patients or patients with hyperdynamic states.

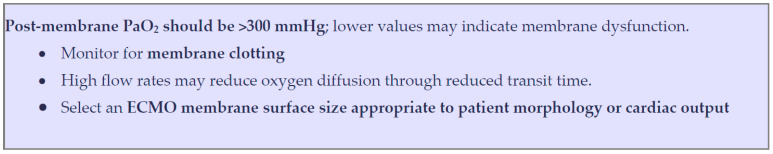



## 5. Standardized Protocol

Initiation of shared guidelines and standardized protocols has been largely associated with higher quality of care [36]. Implementing local protocols for the management of refractory hypoxemia in vvECMO patients could be beneficial. Several studies [37,38] have shown that implementing such guidelines may improve survival, reduce ICU length of stay, and increase ventilator-free days. Zhou et al. [37] conducted a single-center, retrospective cohort study of patients undergoing vvECMO and found that after implanting a local protocol for refractory hypoxemia, compliance with protective ventilation strategies during ECMO support improved. They observed lower mean plateau pressure, driving pressure, and tidal volume during ECMO course. Additionally, there was a statistically significant increase in the number of patients undergoing prone positioning and a significant reduction in the use of high-frequency oscillation ventilation [37]. Gallo de Moraes et al. [38] demonstrated comparable results in non-ECMO ARDS with the evidence of shorter time to prone positioning as well as reduced ICU and hospital length of stay.

## 6. Patient-Centered Strategy

### 6.1. Residual Lung Function

In ARDS, protective ventilation (6 mL/kg of ideal body weight [IBW]) has been shown to improve mortality and increase ventilator-free days [39]. This strategy has thus been widely adopted and recommended by guidelines on ARDS [1,19,40]. The rationale for vvECMO use is therefore to deepen this strategy by applying “ultra-protective ventilation” (3–4 mL/kg of IBW, low plateau and driving pressure, high PEEP, and low respiratory rate (RR) [5,6,7,8,9,10]) to put the lung at maximum rest in order to further improve lung regeneration and potentially improve survival.

Comparison of both ventilation strategies are summarized in Table 2.

Despite the pathophysiological rationale for ultra-protective ventilation, data on its impact on barotrauma or survival outcomes remain controversial [41,42]. Two studies conducted by Li et al. [43] and Nishikimi et al. [44] looking at ultra-protective ventilation in vaECMO and vvECMO might add some evidence to current knowledge. Therefore, optimizing residual lung function by titrating the ventilator to higher PEEP, plateau pressure (PPlat), RR, and FiO_2_ may be necessary, especially in the obese population.

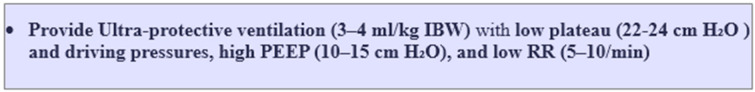



Use of concomitant vvECMO with prone positioning, optimal PEEP with recruitment maneuvers, and utilization of an esophageal probe as well as with inhaled nitric oxide are discussed further.

*Recruitment maneuvers:* Recruitment maneuvers (RMs) are sustained but temporary increases in transpulmonary pressure aimed at reopening collapsed alveoli (incremental increases in PEEP to achieve airway pressures ≥35 cm H_2_O) [1], enhancing the lung volume involved in gas exchange and ultimately improving oxygenation [45]. Their routine use has been controversial in the literature due to concerns about hemodynamic collapse, barotrauma, and ventilator-induced injury [46]. Several methods exist, including the Staircase Recruitment Maneuver (SRM) described in the ART study [47], the sustained inflation recruitment maneuver [48], or a gradual increase in continuous airway pressure (CPAP 40 cm H_2_O) [49]. However, recent meta-analyses of non-ECMO ARDS [45,50,51] failed to prove increased survival. Among obese patients, the PHARLAP study (mean BMI of 31 kg/m^2^) [52] and De Santis Santiago et al. (mean BMI of 57 kg/m^2^) [53] did not demonstrate improved survival despite good hemodynamic tolerance. Although, among survivors, obese patients showed more ventilator-free days and less need for adjunctive therapies (vvECMO and inhaled nitric oxide). Based on this evidence, recruitment maneuvers appear to be a reasonable technique for optimizing ventilation in obese patients who require improvement in their residual lung function.

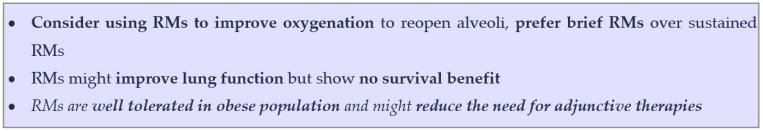



*Higher titration PEEP and use of esophageal probe:* A low driving pressure (less than 15 mmHg) may be associated with lower mortality in ARDS [54]. Ultra-protective ventilation with very low respiratory rates and very low tidal volumes can cause progressive derecruitment of lungs, induce atelectasis, worsen gas exchange, and increase shear stress to the alveoli. To minimize this phenomenon, an open lung strategy has been proposed involving the use of a higher PEEP combined with low tidal volume. This approach leads to low driving pressure and therefore hypoventilation. vvECMO facilitates this strategy by efficiently removing the CO_2_ from the blood. While some animal studies have suggested that ultra-protective ventilation may help to reduce lung inflammation, Deniel et al. [55] showed that low tidal volume, PEEP titrated to a maximum of 20–25 cmH2O of PPlat, and low RR (5/min) were associated with less lung inflammation. Similarly, a randomized controlled trial conducted by Hermann et al. [56] showed that low RR (4–5/min) associated with high PEEP (14-16 cmH2O) was associated with higher ventilator-free days than conventional ventilation. Mortality was lower in the ultra-protective group but did not reach statistical significance.

In obese patients, PEEP is often underestimated [10,57,58], although higher PEEP levels may be necessary due to increased abdominal pressure and chest wall weight, with some studies suggesting a survival benefit [10,52,59]. Moreover, experimental studies have shown that pleural pressure and transpulmonary pressure were not directly correlated with PPlat in the obese population [60,61]. Consequently, esophageal pressure (PES) monitoring has been considered a potential tool in obese and super-obese patients on vvECMO. However, the EPVent-2 study [62] found no superiority of PES monitoring, despite higher PEEP in the PES group and a high prevalence of obesity in their population. In a post hoc reanalysis of the EPVent-2 study [63], the authors demonstrated the lowest mortality when end expiratory transpulmonary pressure was equal to 0 cmH_2_0. Conversely, studies by De Jong et al. [61] and Terry et al. [64] found that in an obese population, elevated driving pressure and plateau pressure were not correlated with higher mortality in ARDS.

Given the lack of certainty, these measures could serve as a valid adjunctive therapy to guide ventilation in the obese vvECMO population. Nonetheless, these practices should be individualized based on patient-specific data, clinical relevance, and institutional practices.

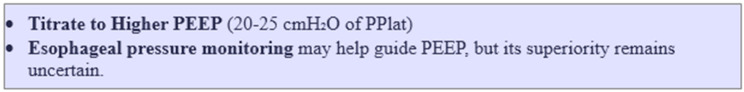



*Prone positioning:* Prone positioning (PP) improves oxygenation by enhancing ventilation–perfusion matching by providing a more homogenous distribution of transpulmonary pressure across the lungs [10]. PP has been demonstrated to increase survival in severe ARDS (when PaO_2_ to FiO_2_ ratio drop under 150) [65], and current ARDS guidelines [1,20,40] strongly recommend PP in these cases. Several studies have shown the feasibility of PP in both obese [66] and ECMO patients [67]. However, evidence supporting the routine use of PP during vvECMO remains controversial. An early retrospective trial by Petit et al. [67] suggested improved survival with PP and vvECMO. Other small retrospective studies showed possible trends toward improved survival with PP, especially if applied early (less than 24 h after cannulation) [68,69]. Additionally, a recent prospective observational trial from patients with non-COVID-19 ARDS conducted by Wang et al. [70] showed improvement in weaning from ECMO and sixty-day mortality. Patients all underwent PP before ECMO implementation. Finally, a pooled individual patient data analysis of European cohort studies conducted by Giani et al. [71] showed improvement in ICU mortality, but this did not reach statistical significance. However, when patients were matched on baseline characteristics using a propensity score, those in the prone group had a lower 60-day mortality. Despite these encouraging findings, the first multicenter prospective study PRONECMO [72] published in 2023 by Schmidt et al. did not demonstrate a significant benefit of PP in ECMO weaning time and mortality. The study involved patients with a mean BMI of 33 kg/m^2^, reflecting the high prevalence of obesity in their population. Several limitations may have influenced the results, including prior use of PP before ECMO initiation, the COVID-19 pandemic, the small sample size, and the study design. Nevertheless, given its safety and feasibility, use of PP during ECMO could still be a viable option for obese patients with low pulmonary compliance.

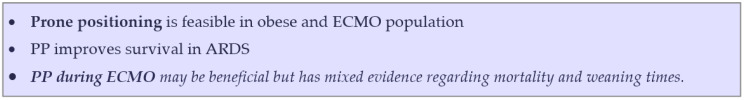



*Inhaled nitric oxide:* Inhaled nitric oxide (InhNO) enhances oxygenation and gas exchange through the dilation of alveolar vessels receiving oxygen, thereby reducing ventilation–perfusion mismatch and improving oxygen capture by ventilated alveoli [73]. This mechanism allows for improved oxygenation without altering ventilator settings, offering an elegant alternative in patients with high ventilator pressures. Despite this encouraging pathophysiological approach, the literature has failed to demonstrate the superiority of InhNO in ARDS [74,75], and a meta-analysis even showed the potential harmful effect of InhNO. The harmful effect of InhNO can be explained by the formation of peroxynitric acid in the situation of high FiO_2_, which is a highly pulmonary toxic substance [76]. As a result, use of InhNO was mostly abandoned. The COVID-19 pandemic, however, renewed interest in the technique, and InhNO was widely used to treat refractory hypoxemia in patients with COVID-19-related ARDS, with studies reporting the use of InhNO in approximately 20–25% of ARDS patients [77,78]. While research in both COVID-19 and non-COVID-19 ARDS showed improved oxygenation, no survival benefit was demonstrated [79,80,81,82]. One study suggested that patients with vvECMO criteria might benefit from InhNO [80], though further research is needed to confirm this finding. In a small recent observational study conducted by Muensters et al. [83], ECMO with InhNO in COVID-19 ARDS was associated with a poorer outcome, but InhNO responders had better survival. Therefore, nitric oxide cannot be routinely recommended to treat vvECMO ARDS with refractory hypoxemia and should be used as a last resort if deemed appropriate.

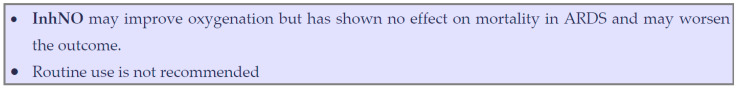



### 6.2. Reducing Oxygen Consumption and Carbon Dioxide Production

One way to minimize tissue hypoxia and improve oxygenation is by reducing the body’s overall oxygen consumption, which can be achieved through simple approaches. Common causes of elevated oxygen utilization (VO_2_) include sepsis, fever, agitation, movements, and shivering. All these causes should be considered in cases of worsening hypoxemia in vvECMO, where other previous complications have been ruled out. Optimizing sedation, favoring a completely controlled ventilation strategy, and ultimately using an intermittent or continuous neuromuscular blockade can help achieve lower oxygen consumption targets. This can also decrease CO_2_ production. Multimodal use of sedation may be necessary in obese patients, as they often require a higher dose of sedatives to obtain the sedation goal. The adsorption or sequestration of sedative drugs within the ECMO circuit further complicates the situation [84]. Sedative agents include morphine derivatives, propofol, benzodiazepines, ketamine, clonidine, and occasionally neuroleptic or anti-epileptic drugs [84]. Fever, although rare in the vvECMO setting, is also a trigger for increased oxygen consumption; consequently, strict temperature control needs to be performed throughout the ECMO circuit.

*Neuromuscular blockade:* The neuromuscular blockade (NMB) plays a nuanced role in the management of ARDS. Neuromuscular blocking agents (NMBAs) have been shown to decrease oxygen consumption by eliminating muscular activity and improving systemic oxygenation, particularly in muscles involved in respiratory function. This mechanism reduces respiratory demand and cardiac output, leading to increased mixed venous oxygen partial pressure and arterial oxygen partial pressure. By reducing the work of breathing during mechanical ventilation, NMBAs can significantly lower whole-body oxygen consumption, with reports of up to a 25% reduction [85]. Additionally, this redistribution of blood flow to the splanchnic and other non-vital vascular beds contributes to sparing oxygen for vital organs. NMBAs may also mitigate ventilator-induced lung injury (VILI) by reducing high minute ventilation and excessive patient effort. However, the impact of NMBAs on mortality remains controversial, especially in the ECMO population, for whom data are lacking. De Grado et al. studied current practice among ECMO patients, and they showed that only 12 percent of ECMO patients receive continuous NMBs [86], emphasizing the uncertainty regarding this practice. Kressin et al. [87] reported no change in ECMO free days, ICU length of stay, or mortality in a small single-center, retrospective observational cohort study involving 47 ECMO patients. In the ECMObesity trial [11], which showed improved survival of obese patients with vvECMO compared to non-obese populations, 68% of obese patients received NMBs compared to 59% of non-obese. No randomized controlled trials exist regarding the use of NMBs in the ECMO population; therefore, current evidence is based on the non-ECMO ARDS population. Key studies have reported conflicting outcomes: the Early Neuromuscular Blockade in the Acute Respiratory Distress Syndrome (ROSE) [88] trial found no survival benefit from early NMBs, whereas the Neuromuscular Blockers in Early Acute Respiratory Distress Syndrome (ACURASYS) [89] trial demonstrated improved survival rates. Neither study provided an indication of BMI in their population, but the ROSE study excluded super-obese patients from their protocol. Finally, a recent meta-analysis reported increased survival among non-ECMO ARDS patients treated with NMBAs [90]. Clinical evidence supporting the use of NMBAs in vvECMO are lacking. Integrating data from non-ECMO ARDS might provide an indication on use in the vvECMO setting in addition to pathophysiological data.

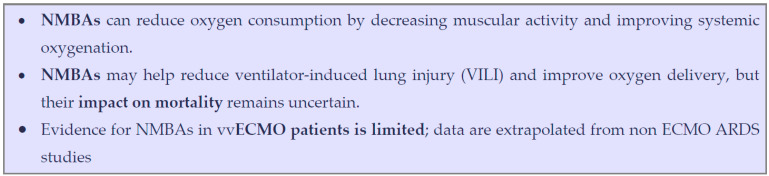



### 6.3. Optimizing Oxygen Delivery and Transfusion Strategy

Hunsicker et al. [91] demonstrated that a restrictive transfusion strategy (<8 g/dL) in ECMO and non-ECMO ARDS patients resulted in similar survival as a liberal strategy (<10 g/dL). For patients with severe ARDS treated with vv-ECMO, the optimal hemoglobin level has not been well defined. The Extracorporeal Life Support Organization (ELSO) guidelines [5] recommend maintaining a normal hematocrit level during ECMO treatment; however, some small retrospective studies [92,93,94] have shown a trend toward similar survival, with a transfusion threshold of 7 g/dL of hemoglobin. Additionally, the PROTECMO observational study [95] showed no sign of improved survival when the transfusion threshold was set higher than 7 g/dL of hemoglobin. Thus, the routine hemoglobin level of a vv-ECMO course could be set at 7g/dL if appropriate tissue oxygenation is provided.

That being said, and on top of previous strategies, in cases of tissue hypoxia despite previous measures ensuring adequate oxygen delivery, higher hemoglobin level and therefore liberal transfusion strategy might be employed reasonably, especially since Schmidt et al. showed improvement in SaO_2_ and PaO_2_ with higher hemoglobin levels [27].

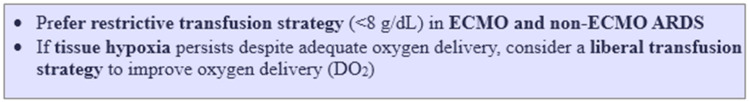



### 6.4. Optimizing ECMO Blood Flow to Patient Blood Flow Ratio (Q_ECMO_/Q_CO_ Ratio)

When managing refractory hypoxemia and when considering vvECMO in cases of ARDS, the clinician should primarily monitor the patient’s CO and calculate the ECMO blood flow to patient’s CO ratio (Q_ECMO_/Q_CO_). The optimal ratio has been shown to be set at >60% [27]. If the ratio is too low (meaning there will be significant shunting into the dysfunctioning patient’s lungs, resulting in hypoxemia), strategies involving optimizing the CO accordingly to ECMO blood flow might be employed.

*General strategy:* The classical approach involves the previous strategies discussed above (optimizing sedation and use of NMBs as decreased metabolic demands often result in decreased CO). If not sufficient, the addition or up titration of clonidine or dexmedetomidine to existing sedation might be employed to benefit from their mechanism of action (stimulating the presynaptic alpha-2 adrenoceptors, thereby decreasing norepinephrine release from both central and peripheral sympathetic nerve terminals) [96] allowing for decreased CO in addition to their sedative effect [97]. Similarly, sinus rhythm should be maintained, and rapid ventricular response atrial fibrillation should be avoided. Vasopressor therapy should be titrated to the minimal acceptable mean pressure to avoid the inotropic effect of norepinephrine and induced arrythmia. Ultimately, cautious use of beta-blockers or, in some cases, therapeutic hypothermia might be considered.

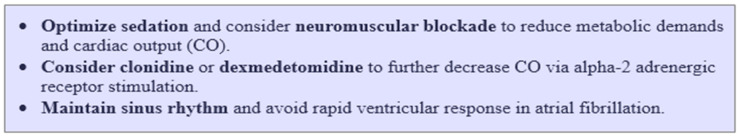



*Beta-blockers:* The rationale for using beta-blockers (BB) is their negative inotropic effect, which can effectively reduce CO and therefore increase the Q_ECMO_/Q_CO_ ratio above 0.6 [27]. Their ability to decrease CO may be of interest in the context of refractory hypoxemia in vv-ECMO due to a low Q_ECMO_/Q_CO_ ratio. Despite these considerations, there are concerns regarding the safety of BB use in this setting.

Since tissue oxygenation (which remains the primary goal when considering vvECMO) ultimately relies on oxygen delivery (DO_2_), BB must demonstrate evidence of improved tissue oxygenation to support their routine use. The pathophysiology regarding BB use in vv-ECMO includes an understanding of the determinants of DO_2_ and arterial oxygen content (CaO_2_).

DO2 and CaO_2_ can be calculated using the following equations:

DO_2_ = CO × CaO_2_

CaO_2_ = 1.34[Hb](SaO_2_/100) + 0.003PaO_2_

These formulas indicate that even if BB may improve SaO_2_ and consequently CaO_2_, by optimizing Q_ECMO_/Q_CO_, they achieve that goal at the cost of a lowered CO, thereby diminishing DO_2_. If high cardiac output is driven by high oxygen demands, a BB-induced reduction in CO is associated with a lower DO_2_.

In their articles, Staudacher et al. [98] and Bommiasamy et al. [99] (in response to Staudacher et al.) demonstrated that despite improving SaO_2_, BB use was associated with lower CO and resulted in decreased DO_2_ in patients with ARDS who where totally dependent on vvECMO for tissue oxygenation. In their original article [98], Staudacher et al. concluded that BB might only be advisable in situations where CO is inappropriately increased and not driven by oxygen demand and that simpler measures, such as discussed above, should be prioritized.

If, despite these concerns, clinicians still decide to use BB, esmolol might be the first-line agent. Esmolol is a short-acting cardio-selective BB administered via continuous infusion. A few small studies have investigated the safety and feasibility of esmolol during the vvECMO course in select tachycardic patients [100,101,102]. They all showed improvements in SaO_2_. Another case series by Kim et al. [103] further confirmed the improvement in SaO_2_ but also the decrease in DO_2_. Krupnik et al. [104] studied the effect of metoprolol in forty-two vvECMO patients with COVID-19 ARDS. They experienced only mild improvement in SaO_2_, and metoprolol was associated with a high rate (47.6%) of adverse events, including hypotension, increased lactate levels, and bradycardia. Therefore, as there is still insufficient to no data to support the routine use of BB in vvECMO in cases of a low Q_ECMO_/Q_CO_ ratio, clinicians should use BB very cautiously. One valid indication could be the use of BB in cases of rapid response supra-ventricular tachycardia.

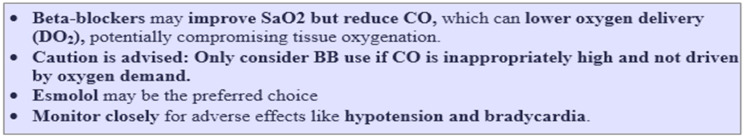



*Therapeutic induced hypothermia:* Hypothermia is associated with bradycardia and low CO with a 7% drop in CO per °C [105]. Considering this, decreasing temperature via the heat exchanger of the ECMO circuit might increase Q_ECMO_/Q_CO_. Moreau et al. [106] summarized the body of evidence in induced hypothermia for high CO patients undergoing vvECMO. The current literature relies on small studies and cases reports, introducing selection and publication biases to the available literature. The optimal target temperature for this population remains unknown due to significant heterogeneity in the available studies. The authors conclude that induced hypothermia is a feasible and relatively safe intervention in the setting of the high CO vv-ECMO population; however, its effects on clinical outcomes remain uncertain.

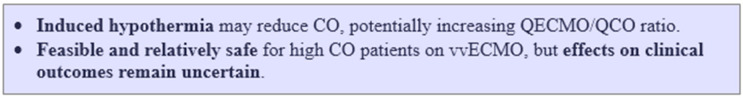



## 7. ECMO Circuit-Centered Strategy

When previous measures become insufficient, modifications to the ECMO circuit can become critical. Modifications involve alterations to the ECMO circuit in order to improve Q_ECMO_/Q_CO_ or to maximize oxygen transfer from the oxygenator to the patient’s blood. Available options include the addition of a third drainage cannula to enhance venous drainage in cases of insufficient ECMO flow, the use of dual oxygenators in parallel or in a series to increase oxygen transfer efficiency, or even transitioning to more complex configurations such as veno-arterio-venous ECMO (v-avECMO) or dual independent (parallel) vvECMO circuits. Although these strategies might seem attractive, they remain purely experimental, as the literature rely only on case series or reports. Such interventions require careful consideration of risks linked to ECMO circuit manipulation, particularly in patients completely dependent on ECMO support. Potential complications include catastrophic hemorrhage, massive air embolism, hemolysis, thrombosis, and mechanical stress on the ECMO system. These measures also place additional pressure on the nursing and/or perfusion teams by increasing the workload required to manage these patients. Given the risks mentioned earlier, these considerations should be implemented in expert centers. However, despite these challenges, such measures can be lifesaving in cases of extreme refractory hypoxemia. When considering these strategies, clinicians should follow a stepwise approach, starting with minimally invasive methods and progressing to more complex ones. Since neither technique has shown clear superiority over the other, the final decision should be based on the clinician’s and ECMO team’s experience, integrating the resources available at their center.

### 7.1. Enhancing Q_ECMO_/Q_CO_ by Employing Large Cannulas or Adding Additional Cannulas

The preferred site of cannulation should be the femoro-jugular configuration, as it decreases the risk of recirculation [33,107] and allows for large-diameter cannulas, effectively providing higher flow. Other cannulation sites include the bi-femoral configuration or supra- or (more rarely) sub-clavian cannulation, which has been described to be a safe alternative in cases of limited venous access [108]. Double lumen cannulation should be avoided in obese patients as it provides only limited flow [107,109,110] (max 4–5 L/min).

Cannula diameters usually range from 17 French to 31 French depending on the manufacturer. Inserting large-diameter cannulas can lead to fear of vessel injury. However, large cannulas are often necessary to achieve high flow in vvECMO [5]. Mauri et al. [111] demonstrated that large-diameter cannulas allowing for a higher ECMO flow were associated with improved survival in COVID-19 ARDS and did not increase the risk of vessel injury. Spinelli et al. [112] further demonstrated positive physiological changes associated with high Q_ECMO_, showing improved SvO_2_, decreased CO, and decreased pulmonary pressure and right ventricle workload, highlighting the need for larger cannulas. Additionally, larger cannulation provides less recirculation [113]. Thus, in the obese population or high-cardiac-output population, it is strongly recommended the cannula diameter be tailored to the patient’s anatomy [109].

A third drainage cannula might be necessary if a high Q_ECMO_ remains difficult to achieve despite the use of a large-diameter cannula. A supplemental cannula can be inserted into the contralateral femoral vein, creating a femoro-femoro-jugular configuration. During the COVID-19 pandemic, two retrospective and observational studies [113,114] demonstrated the feasibility of this technique. This configuration allowed for improved Q_ECMO_ with an identical speed pump but showed no change in post-oxygenator SvO_2_ and no impact on survival.

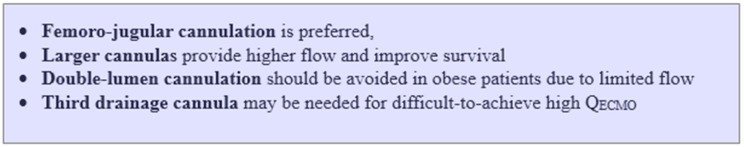



### 7.2. Optimizing ECMO Oxygen Transfer Efficiency

The addition of a second oxygenator, either in parallel or in a series, can improve post-membrane SvO_2_ or PaO_2_. This has only been poorly described in the literature through limited case reports. Miyazato et al. [115] used two oxygenators in parallel in a patient with severe obesity and COVID-19 ARDS. In their case, placement was triggered by persistently low post-membrane PaO_2_ despite several oxygenator changes. The addition of a second oxygenator significantly improved post-membrane PaO_2_ with no further decline, ultimately allowing for vvECMO withdrawal. They demonstrated that a shunt can be induced in the vvECMO circuit if Q_ECMO_ is high, reducing the transit time through the oxygenator and providing insufficient time for oxygen transfer. Hurtado et al. [116] described a three-patient series with severe ARDS secondary to COVID-19 and refractory hypoxemia during ECMO support successfully treated with this approach. On the other hand, Leloup et al. [117] used two oxygenators in a series for refractory hypercapnia in a patient (with a body surface area of 2.77 m^2^) with ARDS following traumatic brain injury. This approach allowed for protective ventilation and restoration of normal PaCO_2_ for brain protection. They did not demonstrate any effect on systemic PaO_2_, as post-membrane oxygenation was already satisfactory. Seadler and al. [118], on their side, conducted an observational study in patients with refractory hypoxemia and hypercapnia on vvECMO during the COVID-19 pandemic using an additional oxygenator placed in parallel. Their approach increased post-membrane PaO_2_ and systemic PaO_2_ while reducing PaCO_2_ and FiO_2_, thereby enabling (ultra)protective ventilation.

In porcine models, Melro et al. [119] studied the efficacy and systemic implications of serial or parallel oxygenators (Jostra–Quadrox D, Maquet, surface membrane: 1.8 m^2^) with max Q_ECMO_ of 5.5 L/min. They showed that both oxygenator configurations resulted in minors changes in circuit blood pressures and systemic oxygenation and modest changes in decarboxylation efficacy. The results observed may be attributed to the lack of data on ECMO flow rates above 7L associated with smaller membrane surfaces, meaning their method could not determine whether it would be effective in this configuration. In their study, they showed that a parallel oxygenator configuration in contrast to the serial configuration offers the advantage of not increasing the ECMO flow resistance. CO_2_ removal was slightly higher in the parallel configuration compared to the serial one. Additionally, Omlor et al. [120] performed an experimental study on a mock ECMO circuit, testing for serial and parallel oxygenation in the context of refractory hypoxemia. In contrast, Melro et al. found that a serial configuration may provide better oxygenation and decarboxylation. They explain this phenomenon using the concept of a “boundary layer”, which is a stationary fluid layer on the outside of the gas fibers that oxygen molecules need to pass via diffusion. The higher blood flow achieved in the serial configuration allowed for thinning of the boundary layer and therefore better oxygen transfer [121]. Thus, the use of two oxygenators in parallel or in a series might offer a safe and relatively simple alternative in cases of refractory hypoxemia induced by shunting through the ECMO circuit when a high Q_ECMO_ is needed.

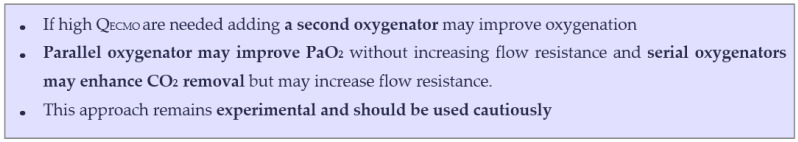



### 7.3. The Use of Additional Circuits

If hypoxemia persists despite previous measures, and Q_ECMO_/Q_CO_ remains below 0.6, an alternative to previous vvECMO modifications is the use of a complete additional circuit, either in series or in parallel. This configuration requires the separation of two independent vvECMOs and necessitates appropriate volemia for additional cannula insertion, as it can lead to severe vascular injury due to the requirement of the placement of two cannulas in the inferior vena cava. Although the parallel configuration is more common (allowing for better improvement in total Q_ECMO_), the serial configuration has also been used. Kang et al. [122] described the use of two serial extra corporeal membrane oxygenators, allowing for weaning of the ECMO support in an obese patient during the COVID-19 pandemic. Several cases reports demonstrated the feasibility of this approach [123,124,125]. Kwon et al. [123] used two parallel vv-ECMO circuits in a burn patient with acute lung injury in order to palliate the high cardiac output of the patient. In their configuration, they used two separate ECMO circuits with two separate drainage cannulas going through two different pumps and oxygenators, which were then reunited in a “Y” shape configuration in the jugular vein. Patel et al. described a case series of five patients treated with parallel vvECMO circuits with relatively successful results [126]. They further demonstrated the effectiveness and feasibility of the approach with four out of five patients surviving until ICU discharge. They also showed that the duration of ECMO support could be prolonged up to 73 days in this configuration without a high rate of complications. Another observational study [127] described the routine use of two parallel vvECMO circuits for refractory hypoxemia in 22 obese patients (mean body weight of 122 kg), further demonstrating the feasibility and need for higher Q_ECMO_ in the obese population with a high CO (mean CO of 14 L/min). Among their cohort, 16 patients out of 22 survived, with a mean ECMO support duration of 40 days. They experienced very few cannulation problems, but their patients required a mean of two changes in ECMO circuit during ECMO support, demonstrating the high workload for these patients. A limitation of their study was that none of their patients underwent PP before or during ECMO support, despite its proven safety and effectiveness in both obese and ECMO populations [65,66,67,68,69,70]. Although Patel et al. [126,127] reported only a few cannulation complications, the technique still requires a high nursing workload, two additional cannulations with two cannulas in the inferior vena cava, and one cannula in each jugular vein, doubling the risk of potential complications. Therefore, while technically feasible, potentially safe, and capable of significantly improving the Q_ECMO_/Q_CO_ ratio, leading to better systemic oxygenation in obese patients with high CO, double-circuit vvECMO should be used as a last resort in experienced centers when other techniques have failed.

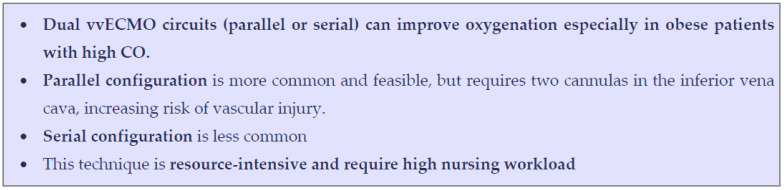



### 7.4. Other Techniques (V-AV, VA, or VV-VA ECMO)

*Switching from vvECMO to vaECMO*: Extracorporeal membrane oxygenation provides a variable content of oxygen to the blood that is directly related to the hemoglobin × blood flow rate. Delivery of that oxygen content to the arterial system achieves little or no increase in systemic oxygen delivery over vvECMO [5], with an increase in meaningful complications [128]. In the case of severe ARDS treated with vaECMO, as the heart recovers, patients can have upper body (and cerebral) hypoxemia; this is known as “Differential hypoxemia”, “Harlequin” [129], or “North/South syndrome”. Therefore, this approach cannot be recommended to manage refractory hypoxemia in high cardiac output patients [5].

*V-AV configuration:* In cases of refractory hypoxemia during vaECMO and the occurrence of Harlequin syndrome due to the association of heart and lung failure, the addition of a jugular return cannula (v-avECMO) might be required [130,131]. V-avECMO allows for bypassing the native lung to oxygenate the upper body. This configuration is only used in cases of concomitant heart and lung failure and cannot be recommended for refractory hypoxemia due to high CO during the vaECMO course due to flow competition, where classical vvECMO would be sufficient.

*More complex strategies:* Navas Blanco et al. [132] described a case of management of refractory hypoxemia with a double va and vvECMO circuit in an obese patient. In their case, due to high CO, requiring high Q_ECMO_, they switched from classical vvECMO to a v-vvECMO configuration. Later, they needed to add a va-ECMO circuit in parallel due to cardiovascular collapse. They justified the use of the VA and VV circuits by the high flow required in their obese patient and the shear stress potentially caused by high flow through the oxygenators and pumps required to provide sufficient Q_ECMO_. The need for this technique may have been driven by the high hemodynamic support required to meet the elevated metabolic demands associated with the patient’s size (BMI of 54 kg/m^2^ and BSA of 3.27 m^2^). Ka et al. [133] also described a case of using a double VV and VA circuit in parallel. Contrary to Navas Blanco et al., their patient was not obese and did not experience a high cardiac output. Their rationale for implementing VV and VA circuits in parallel was driven by the fact that the V-AV configuration would require a temporary pause in ECMO support (risking catastrophic complications) and the low blood flow provided by their machine (maximum 4 L/min). These few case reports cannot provide enough evidence to support the routine use of this configuration in situations of refractory hypoxemia associated with heart failure.

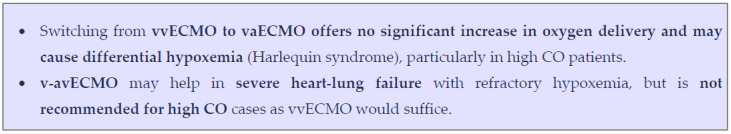



Additional strategies exist, including the use of Impella [134] or Pulmonary artery ECMO (paECMO) [135], mixing support in case of associated heart and lung failure. These associations are outside the scope of this article and are not discussed further.

### 7.5. Resource and Cost Effectiveness

ECMO patients require highly specific needs and an increased nursing workload. Lucchini et al. [136] demonstrated in their study that the nursing activity score (NAS) was significantly higher in the ECMO population and recommended, in alignment with current guidelines, a patient to nurse ratio of 1 with highly trained nurses [137]. The implementation of a second ECMO circuit, whether in parallel or in a series, significantly increased the demand on healthcare resources, particularly in terms of nursing workload [138], perfusionist involvement, and overall medical resource allocation. Standard patient care, including repositioning, hygiene, and routine interventions, becomes exceedingly complex, often necessitating the involvement of multiple nurses or caregivers to safely execute even basic tasks. This added complexity can lead to increased staff fatigue, higher rates of burnout, and a greater risk of complications due to potential lapses in protocol adherence [139].

From a financial perspective, studies evaluating the cost of ECMO demonstrated that patients receiving ECMO had significant higher costs compared to non-ECMO ICU patients [140]. These advanced strategies contribute, consequently, to a marked increase in healthcare costs, primarily due to prolonged ICU length of stay, heightened consumable usage, and the need for multiple ECMO circuit exchanges [127]. Patients with refractory hypoxemia requiring such advanced configurations often experience longer durations of ECMO support, with studies reporting median ECMO runs exceeding 40–70 days in certain cohorts [126]. Each ECMO circuit change represents a substantial financial burden, depending on institutional pricing and regional healthcare policies ranging from tens of thousands to hundreds of thousands of USD [140,141,142]. On average, 82% of costs for the total hospital stay were related to personnel use. Blood products constituted 7%, laboratory and radiology 2.5%, disposable items 3%, and medication 1.5% [141]. Potential complications such as vascular injury and infection further prolong hospital stays, compounding both direct and indirect costs. Finally, in addition to the high costs and significant workload demands, advanced ECMO strategies can exacerbate resource depletion, placing further strain on ECMO-capable centers, especially during periods of heightened demand, such as the COVID-19 pandemic.

## 8. Conclusions

Veno-venous extracorporeal membrane oxygenation has evolved into a crucial intervention for patients with severe ARDS and refractory respiratory failure, particularly in cases where conventional therapies have failed. While obesity presents unique physiological and technical challenges, emerging evidence suggests that it should not be viewed as a contraindication to vvECMO. Contrary to initial concerns, obese and even super-obese patients appear to exhibit comparable or even improved survival rates during ECMO support, reinforcing the concept of the “obesity paradox”.

However, the management of vvECMO in this population requires careful consideration of distinct pathophysiological factors, including increased cardiac output, elevated blood volume, and heightened oxygen consumption, all of which may influence oxygen delivery and ECMO efficiency. Technical challenges such as percutaneous cannulation, infection risk, and anticoagulation strategies must be addressed to optimize patient outcomes.

Given the increasing prevalence of obesity in critical care settings, ICU clinicians must enhance their expertise in vvECMO strategies tailored to this high-risk group. Further studies are needed to refine best practices and determine the optimal management protocols for obese patients requiring ECMO support. Nevertheless, current data indicate that when appropriately managed, vvECMO remains a life-saving intervention that should be strongly considered in the obese population with severe ARDS. However, the implementation of advanced ECMO strategies in this population comes with significant challenges, including increased costs, heightened resource utilization, and a substantial burden on ICU staff, particularly the nursing team, which must be carefully weighed when considering these interventions.

## Figures and Tables

**Table 1:** EOLIA criteria for ECMO initiation [2] and classical contraindications of vvECMO [5].

EOLIA Criteria	Contraindications
PaO_2_ to FiO_2_ ratio < 80 for more than 6 h PaO_2_ to FiO_2_ ratio < 50 for more than 3 h Arterial blood pH < 7.25 PaCO_2_ > 60 mmHg for more than 6 h	Old age
	Significant central nervous system pathology
	Active bleeding
	Contraindication for anticoagulation
*Use of:* FiO_2_ > 0.8 Protective ventilation with tidal volume of 6 mL/kg of PBW, Pplat < 30–32 cmH_2_O, respiratory rate of 35/min PEEP > 10 cmH_2_O Neuromuscular blockade Prone positioning	End-stage lung disease (except if vvECMo is used as a bridge to lung transplant)
	Severe immunosuppression
	Mechanical ventilation with high FiO_2_ (>0.8) for more than seven days

PaO_2_ = Oxygen arterial partial pressure, FiO_2_ = Inspired oxygen fraction, PaCO_2_ = Carbon dioxide arterial partial pressure, PBW = Predicted body weight, Pplat = Plateau pressure, PEEP = Positive end-expiratory pressure.

**Table 2:** Comparison of protective and ultra-protective ventilation strategies.

Ventilation Strategy	Protective Ventilation	Ultra (or Super) Protective Ventilation
***Volume***	6–8 mL/kg of ideal body weight	3–4 mL/kg of ideal body weight
***Positive end expiratory pressure (PEEP)***	>5 cm H_2_O	10–15 cm H_2_O
***Inspired oxygen fraction (FiO_2_)***	Titrated to obtain optimal tissue oxygenation	Optimal FiO_2_ of 0.3
***Max respiratory rate***	35 cycles/min	5 to 10 cycles/min
***Max targeted driving pressure***	<15 cm H_2_O	<15 cm H_2_O
***Max targeted plateau pressure***	28–30 cm H_2_O	22–24 cm H_2_O
***Permissive hypercapnia and acidosis***	PH > 7.30–7.25 and PaCO_2_ < 60 mmHg	Optimal sweeping gas in vvECMO allows normal PaCO_2_ value
